# EGFR-mutant NSCLC progressing on first-line osimertinib: rebiopsy in real-world and impact of second-line therapies (the “Rebiopsy on Osi” study)

**DOI:** 10.1093/oncolo/oyag285

**Published:** 2026-07-25

**Authors:** Eleonora Gariazzo, Francesca Colamartini, Ornella Cantale, Andrea Mogavero, Antonio Vitale, Luca Romano, Marianna Peroni, Erika Rijavec, Marco Banini, Sofia Mazzoni, Mirko Montrone, Giulia Pasello, Antonello Veccia, Francesca Rita Ogliari, Silvana Leo, Diego Cortinovis, Angelo Delmonte, Lorenzo Belluomini, Giulia La Cava, Giulia Meoni, Claudio Sini, Marco Donatello Delcuratolo, Anna Bettini, Maria Pagano, Carlo Genova, Alessandro Russo, Giacomo Pelizzari, Luca Tondulli, Concetta Sergi, Elisa Roca, Chiara Bennati, Salvatore Grisanti, Brigida Stanzione, Sabrina Mariotti, Tiziana Vavalà, Hector Soto Parra, Cinzia Ortega, Daniele Pignataro, Gaetano Lacidogna, Maria Simona Pino, Rita Chiari, Luana Calabrò, Daniele Pozzessere, Silvia Masini, Alain Gelibter, Vieri Scotti, Francesco Grossi, Marcello Tiseo, Emilio Bria, Giulio Metro

**Affiliations:** Medical Oncology, Santa Maria della Misericordia Hospital, University of Perugia, Perugia, PG 06156, Italy; Medical Oncology, Santa Maria della Misericordia Hospital, University of Perugia, Perugia, PG 06156, Italy; Department of Oncology, University of Turin, San Luigi Hospital, Orbassano, TO 10043, Italy; Department of Oncology, University of Turin, San Luigi Hospital, Orbassano, TO 10043, Italy; Fondazione Policlinico Universitario Agostino Gemelli IRCCS, Università Cattolica del Sacro Cuore, Roma, 00168, Italy; Medical Oncology, Santa Maria della Misericordia Hospital, University of Perugia, Perugia, PG 06156, Italy; Department of Medicine and Surgery, University of Parma, Parma, 43121, Italy; Division of Medical Oncology, ASST dei Sette Laghi, Varese, VA 2100, Italy; Department of Experimental and Clinical Biomedical Sciences “Mario Serio,” University of Florence , Florence, FI 51134, Italy; Medical Oncology, Santa Maria della Misericordia Hospital, University of Perugia, Perugia, PG 06156, Italy; Medical Thoracic Oncology Unit, IRCCS Istituto Tumori “Giovanni Paolo II,”Bari, 70124, Italy; Medical Oncology 2, Veneto Institute of Oncology IOV—IRCCS, Padua, PD 35128, Italy; Department of Surgery, Oncology and Gastroenterology, University of Padova, Padua, 35124, Italy; Medical Oncology, Santa Chiara Hospital, Largo Medaglie d‘Oro 1, TN 38122, Italy; Department of Medical Oncology, IRCCS Ospedale San Raffaele, Milan, 20132, Italy; Department of Medical Oncology, “Vito Fazzi” Hospital, Lecce, 73100, Italy; Medical Oncology Unit, Fondazione IRCCS San Gerardo dei Tintori, Monza, MB 20900, Italy; Department of Medicine, University of Milano-Bicocca, Milan, MI 20126, Italy; Department of Medical Oncology, Istituto Romagnolo per lo Studio dei Tumori “Dino Amadori” (IRST), Istituto di Ricovero e Cura a Carattere Scientifico (IRCCS), Meldola, 47014, Italy; Section of Innovation Biomedicine—Oncology Area, Department of Engineering for Innovation Medicine (DIMI), University of Verona and University and Hospital Trust (AOUI) of Verona, 37126, Italy; Medical Oncology, Campus Bio-Medico University, Rome, 00128, Italy; SOS Medical Oncology, San Giovanni di Dio Hospital, Firenze, FI 50143, Italy; Medical Oncology, CPDO, Ospedale Giovanni Paolo II, Olbia, SS 07026, Italy; Medical Oncology Unit, Fondazione IRCCS Casa Sollievo della Sofferenza, San Giovanni Rotondo (FG) 71013, Italy; Medical Oncology, ASST Papa Giovanni XXIII, Bergamo, 24127, Italy; Oncologia Medica IRCCS Arcispedale Maria Nuova Reggio Emilia, Reggio Emilia, 42123, Italy; Academic Medical Oncology Unit, IRCCS Ospedale Policlinico San Martino, Genoa 16132, Italy; Department of Internal Medicine and Medical Specialties, University of Genoa, Genoa 16132, Italy; Medical Oncology Department, Humanitas Istituto Clinico Catanese, Misterbianco, Catania, CT 95123, Italy; Department of Biomedical Sciences, Humanitas University, Pieve Emanuele, 20090, Italy; Department of Oncology, Azienda sanitaria universitaria Friuli Centrale, Udine, UD 33100, Italy; Medical Oncology, San Maurizio Central Hospital, South Tyrolean Healthcare System, Bolzano, BZ 39100, Italy; Oncology Unit, ARNAS Garibaldi Nesima, Catania, 95123, Italy; Thoracic Oncology-Lung Unit, P. Pederzoli Hospital, Peschiera Del Garda (VR), 37019, Italy; Oncology Unit, Ospedale Santa Maria delle Croci, Ravenna, 48121, Italy; Medical Oncology Unit, ASST Spedali Civili di Brescia, University of Brescia, Brescia, BS 25123, Italy; Dipartimento di Oncologia Medica, Centro di Riferimento Oncologico di Aviano (CRO) IRCCS, Aviano 33081, Italy; Medical Oncology, Department of Onco-Hematology, Azienda Ospedaliera Policlinico Tor Vergata, University of Rome Tor Vergata, RM 00133, Italy; AOU Città della Salute e della Scienza, Department of Oncology, SC Oncologia 1U, Torino, TO 10126, Italy; Medical Oncology, Azienda Ospedaliero Universitaria Policlinico “G. Rodolico-S. Marco,”Catania, CT 95123, Italy; Division of Oncology, Institute for Cancer Research and Treatment Asl Cn2-Alba-Brà, CN 12151, Italy; Medical Oncology, Ospedale Cardinal Massaia, Asti, AT 14100, Italy; Medical Oncology, AO Ordine Mauriziano, Turin, TO 10128, Italy; Medical Oncology Unit, Santa Maria Annunziata Hospital, Department of Oncology, Azienda USL Toscana Centro, Florence, FI 50012, Italy; UOC Oncologia AST PU-Pesaro e Fano, Pesaro, PU 61121, Italy; Oncology Unit, University Hospital of Ferrara, Ferrara, FE 44124, Italy; Division of Medical Oncology, S. Stefano Hospital, Azienda USL Toscana Centro, Prato , PO 59100, Italy; Medical Oncology, IRCSS Humanitas Research Hospital, Rozzano, MI 20089, Italy; Department of Radiological Sciences, Oncology and Pathology, Sapienza University of Rome, RM 00161, Italy; Azienda Ospedaliero-Universitaria Careggi, Florence, FI 50134, Italy; Division of Medical Oncology, ASST dei Sette Laghi, Varese, VA 2100, Italy; IRCCS Azienda Ospedaliera Metropolitana, Genova, GE 16132, Italy; Department of Medicine and Surgery, University of Parma, Parma, 43121, Italy; Medical Oncology Unit, University Hospital of Parma, Parma, PR 43126, Italy; Fondazione Policlinico Universitario Agostino Gemelli IRCCS, Università Cattolica del Sacro Cuore, Roma, 00168, Italy; Ospedale Isola Tiberina—Gemelli Isola, Rome, RM 00186, Italy; Medical Oncology, Santa Maria della Misericordia Hospital, University of Perugia, Perugia, PG 06156, Italy

**Keywords:** EGFR-mutant NSCLC, tumor rebiopsy, real-world study, osimertinib resistance mechanisms, post-osimertinib therapy, MET amplification

## Abstract

**Background:**

Although rebiopsy at progression on osimertinib is recommended for patients with advanced EGFR-mutant non-small cell lung cancer (NSCLC), its real-world impact on clinical outcomes remains unclear. *Rebiopsy on Osi* is a multicenter, retrospective study assessing rebiopsy patterns, resistance mechanisms, and their impact on second-line treatment choices and outcomes in routine clinical practice.

**Methods:**

A total of 457 patients with advanced NSCLC harboring a common *EGFR* mutation (exon 19 deletion or L858R) who progressed on first-line osimertinib were identified from the Italian ATLAS registry. Patients were stratified according to rebiopsy status (tissue and/or plasma-based next-generation sequencing) and receipt of biomarker-driven adaptive second-line therapy.

**Results:**

Rebiopsy was performed in 206 patients (45.1%), predominantly via tissue sampling (66.2%). A resistance mechanism was identified in 80 cases (38.8%), with higher detection rates with tissue rebiopsy (46.6%) than with liquid biopsy (13.5%). MET amplification/overexpression emerged as the most frequent actionable resistance mechanism. Among 239 patients treated with second-line therapy, 39 (16.3%) received adaptive treatment, including 29 of 39 patients (74.3%) with MET amplification/overexpression who were treated with a MET-TKI-based regimen. Median progression-free survival (PFS) and overall survival (OS) were longer with adaptive therapy (7.3 and 14.1 months) than with rebiopsy without treatment adaptation (6.5 and 12.2 months) or no rebiopsy (5.1 and 8.2 months).

**Conclusion:**

In real-world practice, rebiopsy identifies a resistance mechanism in slightly more than one third of patients, with actionable MET amplification/overexpression accounting for approximately one fifth of cases. Biomarker-driven adaptive therapy may improve clinical outcomes, supporting the implementation of rebiopsy in routine practice.

Implications for practice The clinical impact of rebiopsy at progression on first-line osimertinib in EGFR-mutant advanced non-small cell lung cancer (NSCLC) remains incompletely defined in real-world practice. Our analysis shows that rebiopsy, although underused across Italian institutions, is feasible and can identify actionable resistance mechanisms, enabling biomarker-driven treatment strategies. Patients receiving adaptive therapy based on rebiopsy findings showed improved outcomes, supporting the integration of rebiopsy into routine clinical decision-making whenever feasible.

## Background

Osimertinib, a third-generation epidermal growth factor receptor (EGFR)-tyrosine kinase inhibitor (-TKI), represents a standard first-line therapy for patients with advanced non–small cell lung cancer (NSCLC) harboring a common *EGFR* mutation, namely exon 19 deletion (ex19 del) or L858R, following the results of the pivotal *FLAURA* trial. In this randomized phase III study, osimertinib demonstrated superior outcomes over a first-generation EGFR-TKI, achieving a median progression-free survival (mPFS) of 18.6 months and a median overall survival (mOS) of 38.6 months.^1^^,^^2^ Notably, indirect comparisons from the same study also suggested improved outcomes with osimertinib compared with second-generation EGFR-TKIs.^3^ However, disease progression ultimately occurs in virtually all osimertinib-treated patients, most commonly due to acquired resistance. Importantly, rebiopsy at the time of progression has unveiled the onset of multiple resistance mechanisms, many of whom can be targeted through biomarker-driven adaptive strategies.^4^ Accordingly, most international guidelines, including those from the European Society for Medical Oncology (ESMO), recommend performing tumor rebiopsy at progression on first-line osimertinib.^5^ In parallel, other research efforts are exploring biomarker-unselected treatment strategies, in which understanding the underlying resistance mechanisms is less critical for the therapeutic choice in the post-osimertinib setting.^6–9^

Therefore, there is an urgent need for real-world data on the feasibility and performance of rebiopsy to better define its true clinical value in routine practice.

However, there are some challenges to this type of research. Heterogeneity in rebiopsy specimens (tissue, plasma, or both) and the use of different locally validated next-generation sequencing (NGS) panels could represent an obstacle to the generalization of results. In addition, second-line therapies based on putative resistance mechanisms identified at rebiopsy are not always easily accessible in routine practice, potentially limiting the clinical impact of rebiopsy on disease outcome. Furthermore, rebiopsy may delay initiation of subsequent treatment and, as an invasive procedure, carries procedural risks. Consequently, the real-world role of rebiopsy and its impact on outcomes of biomarker-driven adaptive strategies remain to be defined.

To address this knowledge gap, we conducted the *Rebiopsy on Osi* analysis, a multicenter, retrospective study including patients with EGFR-mutant advanced NSCLC progressing on first-line osimertinib across several Italian institutions.

## Methods

### Study population

This retrospective, observational, multicenter study included patients with advanced or metastatic NSCLC harboring common *EGFR* mutations (ex19 del or L858R) who experienced disease progression on first-line osimertinib between October 2019 and February 2025. Clinicopathologic and outcome data were extracted from routinely collected cases within the Italian biomarker ATLAS registry. Information on patients with disease progression was collected from 48 Italian healthcare institutions, including academic (*n* = 19) and nonacademic centers (*n* = 29), by trained data managers and/or physicians. All data were transferred to an external database for statistical analysis on September 29, 2025.

Patients were considered eligible if they developed either primary or acquired resistance to osimertinib. According to the Jackman criteria,^10^ primary resistance was defined as disease progression occurring within 6 months of osimertinib initiation without prior documented benefit, whereas acquired resistance was defined as disease progression after prior clinical benefit while receiving osimertinib (either in terms of a partial response or stable disease lasting at least 6 months).

Eligible patients were classified according to whether a rebiopsy (liquid, tissue, or both) had been performed, and whether they received a second-line treatment (at first radiologic progression or after continuation of osimertinib beyond progression).

Patients receiving second-line therapy were further categorized into 3 predefined groups:

1. No rebiopsy before second-line treatment;
2. Rebiopsy performed but no adaptive treatment;
3. Rebiopsy followed by adaptive treatment guided by the identified resistance mechanism.

Patients were classified as receiving adaptive treatment if second-line therapy was selected on the basis of a resistance mechanism identified by rebiopsy. These included patients harboring an actionable molecular alteration (eg, MET amplification or *BRAF* V600E mutation) who received a matched biomarker-driven treatment, as well as patients with histologic transformation requiring a change in treatment strategy, such as transformation to high-grade neuroendocrine cancer (HGNEC) treated with etoposide-containing regimens.

**Figure 1.**
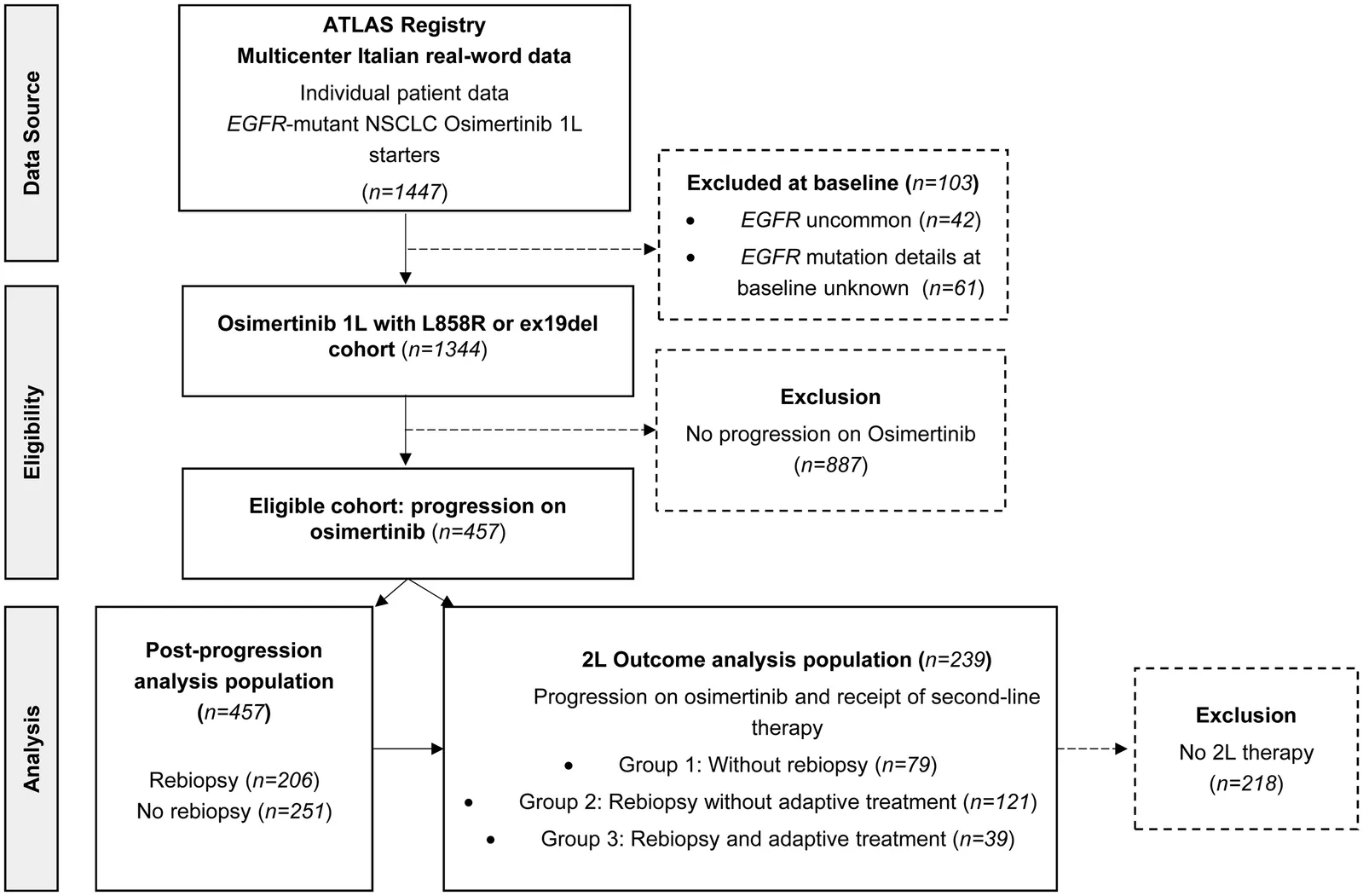
Flowchart depicting patient selection, inclusion and exclusion criteria, and classification of the study population.

Patients’ inclusion and classification workflow, based on the ESMO-GROW guidelines, is shown in Figure 1.

The ATLAS protocol was previously approved by the independent ethics committee of the coordinating center at the University of Turin (ethics approval number: 0006981) and then by the local ethics committees of each participating Center. All patients included in the present study provided written informed consent for inclusion in the ATLAS protocol. The study was conducted in accordance with the International Conference on Harmonization Good Clinical Practice (ICH-GCP) guidelines and the Declaration of Helsinki.

#### Study objectives

The aims of this study were to:

1. Describe the frequency and characteristics of patients undergoing rebiopsy in real-world clinical practice, as well as the type of rebiopsy performed.
2. Summarize rebiopsy findings, with a focus on putative resistance mechanisms.
3. Evaluate the impact of rebiopsy on subsequent treatment selection and clinical outcomes in patients receiving second-line therapy.

### EGFR baseline assessment and rebiopsy performance

Baseline *EGFR* alterations were assessed locally at each participating center using validated assays performed on histologic or cytologic samples. Among rebiopsied patients, post-progression NGS was performed using locally validated and/or commercially available gene panels, and reviewed to document the genomic profile signature at resistance. NGS analyses were accepted for both liquid (plasma) and tissue samples.

### Clinical outcomes

Real-world objective response rate (rwORR) and progression-free survival (rwPFS) were determined via Response Evaluation Criteria In Solid Tumors (RECIST) version 1.1. Assessments by radiologists and/or treating investigators were accepted. PFS was defined as the time from the second-line treatment initiation to documented disease progression or death, whichever occurred first. Patients who did not progress were censored at the time of their last adequate disease assessment. Real-world overall survival (rwOS) was defined as the time from the second-line treatment initiation to death.

### Statistical analyses

Continuous variables were compared using the Wilcoxon-Rank Sum test or Kruskal-Wallis test, as appropriate. Categorical variables were analyzed using Fisher’s exact test. Kaplan-Meier methodology was used to estimate event-time distributions. Log-rank tests were used to assess differences in the event-time distributions, and Cox proportional hazards models were used to generate hazard ratio estimates in univariate and multivariate models. All *P* values were 2-sided and confidence intervals are at the 95% level; statistical significance was set at *P *< .05. Analyses were performed using R version 4.4.3.

## Results

### Patient characteristics and factors associated with rebiopsy

Out of 1344 patients harboring a common *EGFR* mutation and treated with first-line osimertinib, 457 patients experienced disease progression; their characteristics are summarized in Table S1. Briefly, median age was 68 years (range, 27-90) and 66.1% were women. Nearly all patients (97.1%) had adenocarcinoma at initial diagnosis, and 59.5% carried an ex19 deletion at baseline. Prior to progression, 74.3% had achieved an objective response to osimertinib; the mPFS on osimertinib was 14 months (95% CI, 13-17 months). In addition, 47.0% showed multisite progression, and 10.8% had central nervous system (CNS) progression.

We first aimed at identifying the frequency and type of rebiopsy as well as the clinicopathologic characteristics associated with the likelihood of undergoing rebiopsy at the time of progression on osimertinib. In our cohort, 206 patients (45.1% of all progressed patients) underwent rebiopsy (rebiopsy cohort), performed on plasma (liquid biopsy) in 18.4% of rebiopsied cases, on tissue in 66.2%, and on both in 15.4% (Table S2). By contrast, 251 patients (54.9% of all progressed patients) did not undergo rebiopsy (no rebiopsy cohort). Table 1 compares the characteristics of the 2 cohorts. Rebiopsy cohort patients were significantly younger (<70 years old at the time of progression; *P *< .001) and were more likely to harbor an ex19 deletion at baseline (*P *= .008). In addition, they were more likely to have received osimertinib beyond progression (*P *= .025). Second-line treatment was significantly more frequent in the rebiopsy cohort (*P *< .001). Notably, treatment at an academic center was significantly associated with a higher likelihood of rebiopsy (*P *= .044). Other clinical features such as sex, history of tobacco use, histology, type of resistance and progression, CNS progression, and locoregional treatment at progression, were similarly distributed between the 2 groups.

**Table 1.** Comparison of demographic, clinical, and treatment characteristics between patients who underwent rebiopsy and those who did not at progression on first-line osimertinib.

	No rebiopsy (*N* = 251)	Yes rebiopsy (*N* = 206)	*P* value
**Sex at birth**			*.188*
** Female**	173 (68.9%)	129 (62.6%)	
** Male**	78 (31.1%)	77 (37.4%)	
**History of tobacco use**			*.591*
** Ever**	90 (39.1%)	81 (42.2%)	
** Never**	140 (60.9%)	111 (57.8%)	
** Missing**	21	14	
**Age at progression to osimertinib**			*<.001*
** ≥70**	131 (56.2%)	62 (32.6%)	
** <70**	102 (43.8%)	128 (67.4%)	
** Missing**	18	16	
**Histology at diagnosis**			*.565*
** Adenocarcinoma**	240 (96.4%)	200 (98.0%)	
** Squamous**	3 (1.2%)	2 (1.0%)	
** Other**	6 (2.4%)	2 (1.0%)	
** Missing**	2	2	
**EGFR mutations baseline**			*.008*
** Ex19 deletion**	135 (53.8%)	137 (66.5%)	
** L858R**	116 (46.2%)	69 (33.5%)	
**Responder to osimertinib**			*.173*
** Non responders**	69 (28.5%)	44 (22.3%)	
** Responders** ^a^	173 (71.5%)	153 (77.1%)	
** Missing**	9	9	
**Type of resistance** ^b^			.*368*
** Acquired**	203 (86.4%)	182 (89.7%)	
** Primary**	32 (13.6%)	21 (10.3%)	
** Missing**	16	3	
**Type of progression**			*.188*
** Multisite** ^c^	94 (43.7%)	94 (50.8%)	
** Oligoprogression**	121 (56.3%)	91 (49.2%)	
** Missing**	36	21	
**Central nervous system progression**			*.383*
** No**	185 (87.7%)	161 (91.0%)	
** Yes**	26 (12.3%)	16 (9.0%)	
** Missing**	40	29	
**Osimertinib beyond progression**			*.025*
** No**	110 (56.7%)	74 (44.3%)	
** Yes**	84 (43.3%)	93 (55.7%)	
** Missing**	57	39	
**Locoregional treatment at progression**			*.653*
** No**	102 (50.0%)	83 (47.2%)	
** Yes**	102 (50.0%)	93 (52.8%)	
** Missing**	47	30	
**Subsequent systemic therapy**			*<.001*
** No**	172 (68.5%)	46 (22.3%)	
** Yes**	79 (31.5%)	160 (77.7%)	
**Center type**			*.044*
** Non academic**	77 (30.7%)	45 (21.8%)	
** Academic**	174 (69.3%)	161 (78.2%)	

a Responders were defined as patients achieving a complete response (CR) or partial response (PR).

b According to Jackman criteria (Jackman et al, J Clin Oncol 2010).

c Multisite progression was defined as disease progression involving more than 5 sites.

### Rebiopsy results

**Figure 2.**
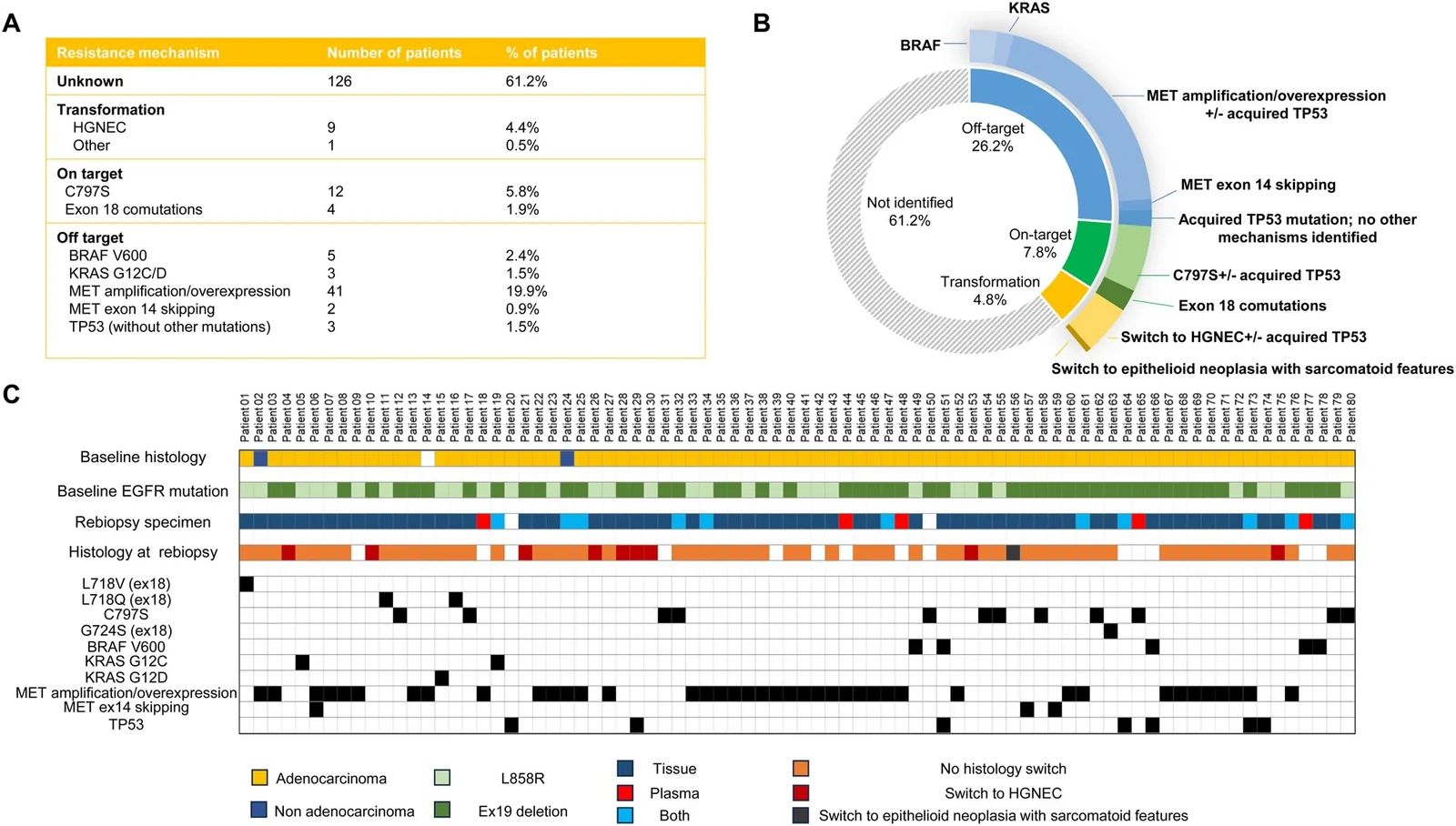
Overview of resistance mechanisms to first-line osimertinib. (A and B) Summary of rebiopsy results in the 206 patients who underwent rebiopsy at the time of disease progression on osimertinib. (C) Oncoprint illustrating clinical and molecular features of the 80 patients in whom a resistance mechanism was identified through rebiopsy.

We next investigated the resistance mechanisms identified through rebiopsy. In slightly more than one third of rebiopsied patients (38.8%), new genomic or histologic alterations were detected. A histologic transformation was observed in 10 patients (4.9%), including 9 cases of HGNEC (8 small cell lung cancers and 1 large cell neuroendocrine carcinoma) and 1 case of epithelioid neoplasia with sarcomatoid features. Among the remaining patients, both on-target (eg, C797S and *EGFR* exon 18 mutations) and off-target alterations (including MET overexpression/amplification, MET ex14 skipping mutations, and mutations in *BRAF*, *KRAS*, or *TP53*) were identified (Table S3; Figure 2A-C).

**Figure 3.**
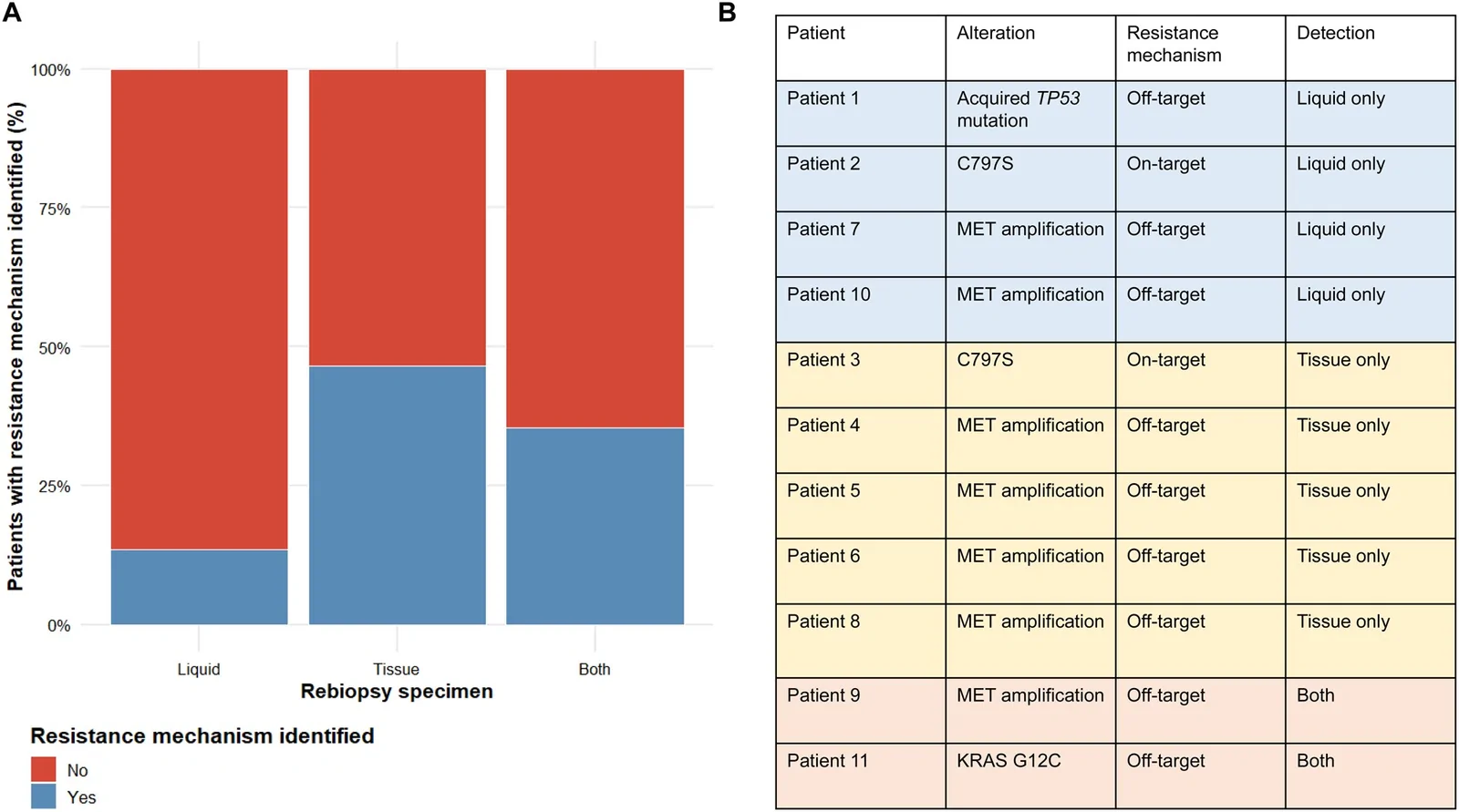
Distribution and concordance of rebiopsy results according to specimen type. (A) Rebiopsy positivity stratified by sample type (tissue, liquid, or both). (B) Patient-level distribution of resistance mechanisms identified in patients who underwent both tissue and liquid biopsy, highlighting the specific alterations and their site of detection (tissue-only, liquid-only, or both).

We also assessed the impact of the rebiopsy specimen type on the detection of resistance mechanisms. In our cohort, a resistance mechanism was identified in 46.6% of the 133 patients who underwent tissue biopsy alone, but only in 13.5% of the 37 patients tested through liquid biopsy alone. Among the 31 patients who underwent both liquid and tissue rebiopsy, 35.5% had an identifiable resistance mechanism (Figure 3A). Overall, resistance mechanisms were detected significantly less frequently in patients undergoing liquid biopsy only compared with those with tissue or combined rebiopsies (*P *= .001), suggesting that plasma genotyping alone may underestimate resistance events detectable in tissue. Among the 11 patients who underwent both liquid and tissue rebiopsies and had a detectable resistance mechanism, the alteration was identified exclusively in tissue in 5 cases (45.5%), exclusively in plasma in 4 cases (36.4%), and in both specimens in 2 cases (18.2%), highlighting only partial concordance between the 2 approaches (Figure 3B).

**Figure 4.**
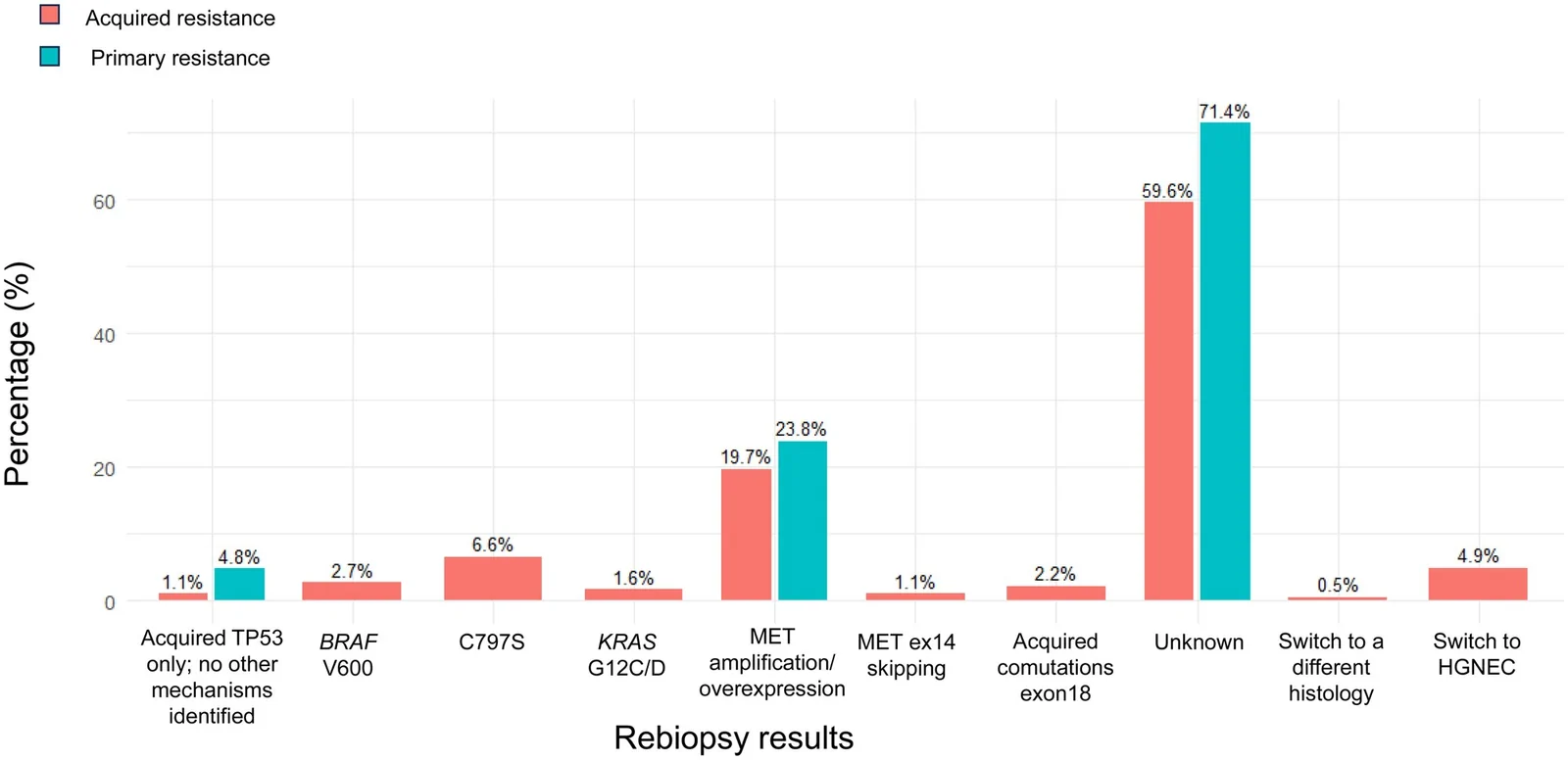
Resistance mechanisms identified through rebiopsy, stratified by type of resistance (primary vs acquired).

Because our cohort included patients with both primary and acquired resistance to osimertinib, as per the Jackman criteria,^10^ we explored whether certain mechanisms were more frequent in a specific type of resistance. In the subset of rebiopsied patients, primary resistance was associated with a higher rate of unknown resistance mechanisms compared with acquired resistance (71.4% vs 59.6%), with MET amplification/overexpression and *TP53* mutations representing the only identifiable mechanisms in cases of primary resistance (Figure 4).

### Rebiopsy impact on second-line treatment choice and clinical outcome

We next investigated whether undergoing rebiopsy at the time of progression had an impact on the choice of second-line therapy. In our cohort, 239 patients received second-line therapy, either immediately after progression or following treatment beyond progression with osimertinib. Table S4 reports the characteristics of patients treated with second-line therapy. The median age at the time of progression was 65 years (range, 27-85), 65.7% were female, 98.8% were White, 80.5% had an ECOG PS of 0-1, and 54.6% experienced multisite progression. Median follow-up from the initiation of second-line treatment was 23.3 months (95% CI, 19.9-not reached). Table 2 lists the type of second-line therapies received according to rebiopsy (yes vs no), further classifying them according to whether rebiopsied patients had received an adaptive treatment linked to a specific putative mechanism of resistance. Among the 160 patients who underwent rebiopsy prior to second-line therapy, 63 were found to have an actionable molecular alteration or histologic transformation. Of these, 39 patients (61.9%) received an adaptive second-line treatment based on the rebiopsy findings, corresponding to 16.3% of the overall second-line cohort (Tables S4, 2) Among patients who received rebiopsy and adaptive therapy, the most frequent resistance mechanism was MET amplification/overexpression, leading to MET-targeted therapy in 29 patients. This was followed by histologic transformation to HGNEC, which led to histology-specific chemotherapy in 7 cases. In contrast, among rebiopsied patients without adaptive treatment, more than half had no identifiable resistance mechanism. Notably, despite the presence of actionable findings, 10 patients with MET amplification/overexpression and 1 patient with *BRAF* V600E mutation were treated with second-line platinum doublet or monochemotherapy. Overall, in the cohort of patients who received a second-line treatment, the ORR to this treatment was 40.7%, the median rwPFS was 6.2 months, and the median rwOS was 11.0 months (Figure S1A-C).

**Table 2.** Distribution of second-line treatment strategies according to rebiopsy status and resistance mechanisms identified at progression on first-line osimertinib. Patients were grouped according to whether rebiopsy was performed and whether the results guided the selection of a matched adaptive treatment.

2nd line treatment group	Resistance mechanism	Treatment	Number of patients
**Rebiopsy and adaptive treatment (*n* = 39)**	BRAF V600E	BRAFi ± MEKi	2
	Histologic transformation to HGNEC	Platinum + VP16 +/- anti-PD-(L)1	7
	MET amplification/overexpression	MET-TKI therapy clinical trial	28
		Tepotinib + osimertinib	1
	On-target G724S mutation	Afatinib	1
**Rebiopsy without adaptive treatment (*n* = 121)**	None identified	Platinum based doublet +/- antiPD-(L)1	70
		Monochemotherapy	3
		Amivantamab-based regimens	8
		Other/non available	13
	BRAF V600E	Platinum based doublet	1
	KRASG12D	Other	1
	MET amplification/overexpression	Platinum based doublet	9
		Monochemotherapy	1
	On-target exon 18 comutations (L718Q, L718V)	Platinum based doublet	2
		Amivantamab-based regimens	1
	On-target C797S comutation	Platinum based doublet	3
		Amivantamab-based regimens	3
		Other/non available	4
	Acquired TP53	Other/non available	2
**No rebiopsy (*n* = 79)**		Platinum based doublet+/- antiPD-(L)1	53
		Monochemotherapy	8
		Amivantamab-based regimens	2
		Other/non available	16

Abbreviations: anti-PD-(L)1, programmed death receptor-1/programmed death ligand-1 inhibitor; BRAFi, BRAF inhibitor; HGNEC, high-grade neuroendocrine carcinoma; MEKi, MEK inhibitor; MET-TKI, MET tyrosine kinase inhibitor; VP16, etoposide.

**Figure 5.**
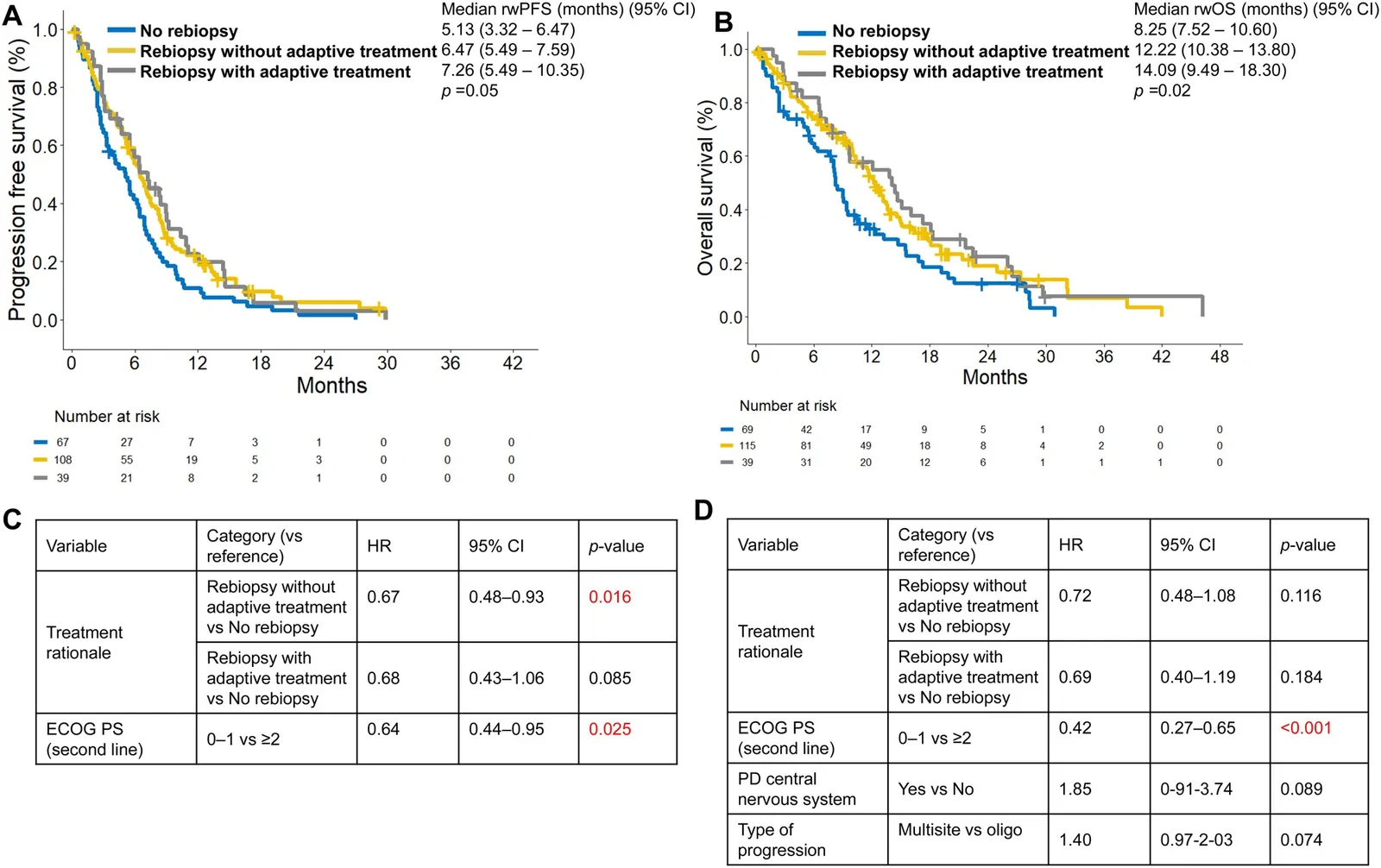
Second-line clinical outcomes stratified by whether rebiopsy findings guided the choice of subsequent therapy. (A) Real world progression-free survival (rwPFS) and (B) real world overall survival (rwOS) from the initiation of second-line treatment, according to 3 groups: no rebiopsy, rebiopsy without an adaptive treatment, and rebiopsy followed by an adaptive treatment. (C) Multivariable Cox proportional hazards model for PFS and (D) multivariable Cox proportional hazards model for OS, including variables that were significant at univariable analysis.

We next investigated whether adaptive treatment according to the putative mechanisms of resistance identified had an impact on clinical outcomes in patients treated with second-line therapy. We therefore stratified patients treated with second-line therapy according to whether they had received an adaptive treatment based on rebiopsy results, no adaptive treatment despite rebiopsy, or no rebiopsy. A stepwise improvement across the 3 groups was observed, with rebiopsied patients who received adaptive therapy showing the highest rwORR (59.5% vs 35.0% vs 39.0%, respectively; *P *= .03), as well as the longest median rwPFS (7.26 vs 6.47 vs 5.13 months, respectively; *P *= .05) and median rwOS (14.09 vs 12.22 vs 8.25 months, respectively; *P *= .02) (Figures S2A and 5A and B). However, after adjustment in multivariable models including variables that were significantly associated with rwPFS and rwOS in univariable analyses (Tables S5 and S6), the differences between treatment groups were no longer statistically significant (Figure 5C and D). Among the variables retained in the models, ECOG PS remained independently associated with both rwPFS and rwOS. Because the group of rebiopsied patients who received adaptive treatment included cases of histologic transformation to HGNEC, who typically have poorer prognostic features, we repeated the analyses after excluding patients with documented switch to HGNEC, in order to minimize potential bias from histologic heterogeneity. After this restriction, the improvement in terms of rwORR (Figure S2B), rwPFS and rwOS became even more pronounced (Figure S3A and B). However, while the multivariable model for rwPFS retained statistical significance, the multivariable model for rwOS showed only a consistent trend without reaching significance (Figure S3C, D).

To further explore the specific impact of treatment adaptation, we performed an additional analysis restricted to patients in whom rebiopsy identified an actionable resistance mechanism or histologic transformation. Within this subgroup, patients who received an adaptive second-line treatment showed numerically higher response rates and longer survival outcomes compared with those who did not receive tailored therapy. However, given the limited sample size of the non-adaptive treatment group, these differences did not reach statistical significance and should be interpreted with caution (Figure S4A-C).

## Discussion

Although several studies, including recent retrospective and prospective analyses, have investigated the role of rebiopsy in patients with EGFR-mutant (ex19del or L858R) NSCLC in routine clinical practice, important knowledge gaps remain. Previous reports were frequently limited by single-center designs and predominantly academic settings,^11^ included patients receiving osimertinib beyond the first-line setting,^12^^,^^13^ or provided limited information on the impact of rebiopsy findings on subsequent treatment selection.^14^ Our study addresses these gaps by leveraging the large multicenter ATLAS registry to evaluate the feasibility of rebiopsy in both academic and nonacademic institutions, characterize resistance mechanisms at progression on first-line osimertinib, and assess the impact of rebiopsy results on second-line treatment decisions and clinical outcomes in a homogeneous population of patients with common *EGFR* mutations.

In this study, we found that slightly less than half of all patients who failed first-line osimertinib underwent any type of a rebiopsy (45.1%, 206 of 457 patients). Interestingly, only a trend toward a higher rebiopsy rate was observed in patients with multisite progression compared with oligoprogression (50.8% vs 43.7%). However, the finding that no more than 50% of multisite progressing patients underwent rebiopsy is not fully aligned with current ESMO guidelines, which recommend liquid and/or tissue rebiopsy in cases of systemic progression on osimertinib.^5^ Our observation is likely driven by the adherence to the ATLAS registry of multiple nonacademic healthcare institutions and might reflect the fact that NGS for liquid and/or tissue rebiopsy may not be easily accessible in centers with little technical and financial resources. Therefore, we believe it is important to promote rebiopsy in patients with systemic progression on osimertinib, particularly in nonacademic centers, potentially through referral pathways to academic institutions. Nevertheless, we also acknowledge that several factors may have contributed to the limited use of tissue rebiopsy in routine practice, including disease location, procedural risks, and deterioration of the clinical condition at the time of progression. Importantly, patients experiencing clinical deterioration may be less likely to undergo rebiopsy, as clinicians may be reluctant to pursue additional invasive procedures, enroll such patients in clinical trials, or initiate further systemic therapy. Consistently, we also found that the rebiopsy cohort included a significantly higher proportion of patients who received second-line therapy, mirroring the fact that rebiopsy may influence physicians’ decision to initiate subsequent systemic treatment.

When analyzing resistance mechanisms overall, we found that 4.9% of cases (9 of 206) underwent histologic transformation to HGNEC. Histologic transformation is a key mechanism of resistance to EGFR-TKIs, which, at present, can be identified only through tissue rebiopsy. In our study, 81.6% of patients (164 of 206) underwent tissue rebiopsy, either alone or sequential to liquid biopsy. Accordingly, the frequency of HGNEC transformation among patients with available tissue samples was 5.5% (9 of 164). By contrast, other series have reported higher rates of histologic transformation, reaching up to 15%.^11^^,^^15^^,^^16^ However, the present analysis is based on one of the largest cohorts of patients who underwent tissue rebiopsy at progression on first-line osimertinib. Therefore, the observed 5.5% rate likely represents a reliable estimate of the real-world incidence of histologic transformation in this clinical setting, further supporting the prioritization of tissue rebiopsy over liquid biopsy to capture this phenomenon.

*EGFR* C797S was the most frequent second-site resistance mutation, identified in 5.8% of patients (12 of 206). This estimate is informative because it derives from a cohort of patients uniformly pretreated with first-line osimertinib. In a recent study, the incidence of EGFR C797S was 19% across patients receiving osimertinib in any treatment line, decreasing to 11% when restricted to those progressing on first-line osimertinib.^13^ Taken together, these data suggest that second-site *EGFR* mutations are considerably less common after upfront osimertinib. This factor should be considered in the design of upcoming clinical trials of fourth-generation EGFR inhibitors in the post-osimertinib setting.

Notably, a substantial proportion of patients exhibited MET amplification as a bypass resistance mechanism, identified in 19.9% of cases (41 of 206, Table S3). In tumor tissue, MET amplification assessed by FISH with a gene copy number ≥5 was reported in 10.1% of patients (21 out of 206). Similarly, a recent study found a 9.4% incidence of MET amplification (gene copy number ≥5) using tissue NGS at progression on first-line osimertinib.^17^ Importantly, the optimal cutoff for MET amplification remains to be clearly defined. While recent studies have restricted MET TKI-based strategies after progression on third-generation EGFR-TKIs to patients with higher-level amplification (eg, FISH ≥10), systematic assessment of MET amplification at progression should be encouraged, as it is increasingly recognized as a key predictive biomarker for MET-targeted therapies.^18–21^ Consistently, in our study, documentation of MET amplification enabled adaptive MET TKI-based treatment in 18% of rebiopsied patients (29 of 160) who received second-line therapy (Table 2). By contrast, the role of MET overexpression as a predictive biomarker remains more uncertain, both in terms of predictive value and optimal positivity thresholds.^19^^,^^22^^,^^23^ In our study, MET IHC 3 + (≥ 50%) was reported in 8 patients, with concurrent MET amplification (GCN ≥5) in 2 cases (Table S3).

Notably, the resistance landscape observed in our cohort was largely consistent with that reported in previous retrospective and prospective studies, with MET amplification, *EGFR* C797S mutations, and histologic transformation representing the most frequent acquired resistance mechanisms.^11–13^ However, the proportion of patients in whom a putative resistance mechanism was identified was somewhat lower than that reported in selected series. This may reflect differences in molecular testing platforms and routine clinical practice, potentially limiting the detection of specific alterations such as *EGFR* amplifications, frequently reported by Choudhury et al,^11^ or MTAP deletions, highlighted in the prospective study by Piotrowska et al.^14^

Overall, a putative resistance mechanism to osimertinib was identified in 38.8% of cases (80 of 206). As expected, this proportion increased to 46.6% when restricting the analysis to patients who underwent tissue rebiopsy alone (62 of 133). These findings are consistent with a previous study limited to post-osimertinib tissue rebiopsies, which reported resistance mechanisms in 52.6% of cases.^11^ Collectively, these observations support the value of tissue rebiopsy whenever clinically feasible, particularly when comprehensive characterization of resistance mechanisms is required.

Nevertheless, liquid biopsy remains an important complementary tool in routine practice owing to its favorable safety profile, feasibility in patients with difficult-to-access disease sites, ability to capture tumor heterogeneity, and suitability for longitudinal monitoring. Previous studies have demonstrated acceptable concordance with tissue-based analyses and adequate sensitivity for detecting specific resistance alterations, including *EGFR C797S* mutations.^24^ However, liquid biopsy remains less effective at identifying certain resistance mechanisms, particularly MET aberrations and histologic transformation to HGNEC.^25^

At present, whether primary and acquired resistance to EGFR-TKIs arise through distinct biological mechanisms remains unclear. In our cohort, EGFR-dependent alterations (including *EGFR* C797S and acquired exon 18 co-mutations) and histologic transformation were observed only in acquired resistance, whereas primary resistance was characterized exclusively by bypass signaling activation (eg, MET amplification/overexpression) and downstream pathway alterations such as TP53 mutations. These findings support the hypothesis that primary resistance to osimertinib could be driven by the prevalence of dominant tumor clones harboring downstream pathway activating alterations associated with poor prognosis.^26^ Consistently, another study found that the selection of clones harboring downstream pathway activating alterations (PIK3C2G, STK11, and EPAS1) drove resistance in 5 patients with paired pre- and post-treatment liquid biopsies and poor clinical outcomes on osimertinib.^27^ Even more interestingly, the presence of de novo MET aberrations has been recently addressed in a randomized phase II trial comparing first-line osimertinib vs osimertinib plus savolitinib as first-line treatment in patients harboring both *EGFR* mutation and MET amplification/overexpression.^23^ The promising results obtained with the addition of savolitinib (mPFS 19.6 vs 9.3 in the control arm) further support the hypothesis that MET aberrations may be responsible for some cases of primary resistance and should be appropriately addressed. Further studies incorporating deeper comprehensive genomic profiling are warranted to clarify whether primary and acquired resistance mechanisms truly differ.

In the present study, out of 239 patients treated with second-line therapy, 66.9% (*n* = 160) had any type of rebiopsy, and approximately one fourth of these (39 of 160, 24.3%) received an adaptive second-line therapy based on the putative mechanisms of resistance to first-line osimertinib. Of note, MET amplification/overexpression was the most common reason for receiving an adaptive second-line therapy with a MET-TKI-based regimen, mostly in the context of a clinical trial. By contrast, 10 of 160 rebiopsied patients (6.2%) had a documented MET amplification/overexpression, but were not treated with a MET-TKI-based therapy, suggesting the importance of implementing research strategies through clinical trials for early access to potentially innovative drugs for eligible patients.

In the present study, we observed that among patients who received second-line therapy, those who underwent rebiopsy and were treated with an adaptive regimen based on putative resistance mechanisms, experienced improved clinical outcomes compared with rebiopsied patients who did not receive adaptive therapy and those who did not undergo rebiopsy. These findings extend prior observations from academic cohorts, including a study by Choudhury et al., in which biomarker-guided second-line treatment was associated with improved outcomes compared with non-adaptive therapy.^11^ In our real-world cohort, adaptive therapy was associated with a median rwPFS and rwOS of 7.3 and 14.1 months, respectively. These outcomes further improved to 8.5 and 15.1 months when patients with histologic transformation to HGNEC were excluded from the clinical outcome analysis, highlighting the particularly poor prognosis associated with this resistance pattern. Consistently, a recent study reported a mOS of only 11 months in patients with small-cell transformation after osimertinib.^28^ Nevertheless, these findings should be interpreted with caution. In our study, patients in the 3 treatment groups differed across several baseline characteristics, including age and *EGFR* mutation subtype, which may have contributed to the different prognosis. Furthermore, although rebiopsy-guided adaptive treatment was associated with improved outcomes in unadjusted analyses, the observed benefit was attenuated in multivariable models, while ECOG PS remained a strong independent predictor of both rwPFS and rwOS. Therefore, our results are hypothesis-generating and should not be interpreted as evidence of a causal effect of rebiopsy or adaptive treatment strategies. Rather, they suggest that, within a real-world population, patients selected for rebiopsy and biomarker-guided treatment may achieve favorable outcomes, supporting further prospective evaluation of this approach.

Notably, clinical outcomes with adaptive therapy compared favorably with those reported for real-world platinum-pemetrexed chemotherapy, the second-line standard of care at the time of our analysis, which yielded mPFS and mOS of 4.5-5.3 and 9.6-13 months, respectively.^29^^,^^30^ Similar PFS outcomes have also been reported with chemotherapy-based strategies combined with continued EGFR blockade, including platinum-pemetrexed plus osimertinib in COMPEL (median PFS, 8.4 months) and platinum-pemetrexed plus amivantamab in MARIPOSA-2 (median PFS, 6.3 months).^6^^,^^31^ Notably, these trials enrolled clinically selected populations, whereas our cohort included patients with ECOG PS ≥2 (19.5%) and CNS progression (8.7%).

We acknowledge that this study has some limitations. First, approximately one fifth of patients underwent liquid biopsy only, which may have led to an underestimation of resistance mechanisms due to insufficient circulating tumor DNA shedding and the inability to assess histologic transformation. Second, different NGS panels with variable genomic coverage were used for liquid and/or tissue rebiopsy, according to local validation at each participating center, potentially affecting the consistency of comprehensive genomic profiling at resistance. In addition, given the retrospective and observational nature of the study, PFS estimates for second-line therapies in the post-osimertinib setting should be interpreted with caution and warrant prospective validation. In particular, it cannot be excluded that patients undergoing rebiopsy were selected based on more favorable prognostic features, allowing sufficient time to perform rebiopsy and await results before initiating second-line treatment.

Nevertheless, this study reflects real-world patterns of patient management and captures outcomes as assessed in routine clinical practice. Moreover, the analysis focuses on a population homogenously pretreated with first-line osimertinib, providing relevant insights into a commonly encountered clinical scenario. At the same time, we acknowledge that first-line treatment for patients with common *EGFR* mutations (ex19 del or L858R) is rapidly evolving, with novel combination strategies recently entering clinical practice. In fact, with the increasing use of first-line combination regimens such as osimertinib with platinum-pemetrexed and lazertinib with amivantamab, now considered among the standard of care based on overall survival improvements versus osimertinib monotherapy, resistance mechanisms at progression might be affected.^32^^,^^33^ However, not all patients will be candidates for upfront combination strategies in the routine clinical practice setting (eg, elderly, frail, or oligometastatic patients). Therefore, osimertinib monotherapy is likely to remain a standard first-line option for a non-negligible proportion of patients with EGFR-mutant NSCLC in real-world practice, and this study may provide relevant information for the management of progression in this context.

In conclusion, this study shows that rebiopsy (liquid and/or tissue) at progression on first-line osimertinib in patients with *EGFR*-mutated NSCLC (ex19 del or L858R), although performed in fewer than half of patients, is feasible in real-world clinical practice. Rebiopsy identified putative resistance mechanisms in approximately one third of patients and informed the selection of biomarker-guided second-line treatment strategies in approximately one fourth. Although patients receiving adaptive treatment achieved encouraging clinical outcomes, the observational nature of this study precludes conclusions regarding causality, highlighting the need for prospective validation of rebiopsy-guided treatment approaches.

## Supplementary Material

oyag285_Supplementary_Data

## Data Availability

Queries for any data not included in the article or its Supplementary files may be directed to the corresponding author.
