# Supplementary material for: EGFR-mutant NSCLC progressing on first-line osimertinib: rebiopsy in real-world and impact of second-line therapies (the “Rebiopsy on Osi” study)
Source: Oncologist. 2026 Jul 25;31(9):oyag285. doi: 10.1093/oncolo/oyag285 (PMC13529361; doi:10.1093/oncolo/oyag285)
Supplement: Supplementary file 1 [file oyag285_supplementary_data.zip › Supplementary_figure_legend.docx]

**Supplementary figure legend**

**Supplementary Figure S1. (A)** Real world objective response rate (rwORR), **(B)** real world progression-free survival (rwPFS), and **(C)** real world overall survival (rwOS) in the entire cohort of patients who received second-line treatment.

**Supplementary Figure S2**. Real-world objective response rate (rwORR) to second-line therapy stratified according to whether rebiopsy findings guided the choice of subsequent treatment. (A) rwORR in all patients who received second-line therapy. (B) rwORR after exclusion of patients with high-grade neuroendocrine carcinoma (HGNEC).

**Supplementary Figure S3.** Second-line clinical outcomes stratified according to whether rebiopsy findings guided the choice of subsequent therapy, after exclusion of patients with high-grade neuroendocrine carcinoma (HGNEC). **(A)** Real world progression-free survival (rwPFS) and **(B)** real world overall survival (rwOS) from the initiation of second-line treatment, according to three groups: no rebiopsy, rebiopsy without an adaptive treatment, and rebiopsy followed by an adaptive treatment.

**Supplementary Figure S4.** Second-line clinical outcomes among patients with actionable resistance mechanisms identified at rebiopsy. (**A**) Objective response rate (ORR), (**B**) real-world progression-free survival (rwPFS), and (**C**) real-world overall survival (rwOS) from initiation of second-line treatment, according to receipt of an adaptive treatment strategy targeting the identified resistance mechanism.
