# Supplementary material for: EGFR-mutant NSCLC progressing on first-line osimertinib: rebiopsy in real-world and impact of second-line therapies (the “Rebiopsy on Osi” study)
Source: Oncologist. 2026 Jul 25;31(9):oyag285. doi: 10.1093/oncolo/oyag285 (PMC13529361; doi:10.1093/oncolo/oyag285)
Supplement: Supplementary file 1 [file oyag285_supplementary_data.zip › Supplementary Tables.docx]

**Supplementary Table S1.** Baseline characteristics of patients with common *EGFR*-mutant NSCLC who developed progression during first-line osimertinib treatment.

|  | N=457 |
| --- | --- |
| Age at the time of progression, median (range) | 68 years (27-90 years) |
| Sex   - Female - Male | 302 (66.1%) 155 (33.9%) |
| History of tobacco use   - Ever - Never - Not available | 171 (40.5%)  251 (59.5%)  35 |
| Ethnicity   - White - Asian - Not available | 324 (98.5%)  5 (1.5%)  128 |
| Histology at diagnosis   - Adenocarcinoma - Squamous - Other - Not available | 440 (97.1%)  5 (1.1%)  8 (1.8)  4 |
| *EGFR* mutation at baseline   - Ex19 deletion - L858R | 272 (59.5%)  185 (40.5%) |
| Responders to osimertinib   - Non responders - Responders^[1]^ - Not available | 113 (25.7%)  326 (74.3%)  18 |
| Median progression free survival on osimertinib, (95% CI) | 14 months (13 – 17 months) |
| Type of resistance^[2]^   - Acquired - Primary - Not available | 385 (87.9%)  53 (12.1%) 19 |
| Type of progression   - Multisite^[3]^ - Oligoprogression - Not available | 188 (47.0%)  212 (53.0%)  57 |
| Central nervous system progression   - No - Yes - Not available | 346 (89.2%)  42 (10.8%)  69 |
| Osimertinib beyond progression   - No - Yes - Not available | 184 (51.0%) 177 (49.0%) 96 |
| Center type   - Non academic - Academic | 122 (26.7%) 335 (73.3%) |

**Supplementary Table S2.** Baseline characteristics of patients who were rebiopsied after progressing to first-line osimertinib.

|  | N=206 (%) |
| --- | --- |
| Age at the time of progression, median (range) | 64 (27-83) |
| Sex   - Female - Male | 129 (62.6%) 77 (37.4%) |
| History of tobacco use   - Ever - Never - Not available | 81 (42.2%)  111 (57.8%)  14 |
| Ethnicity   - White - Asian - Not available | 140 (98.6%)  2 (1.4%)  64 |
| Histology at diagnosis   - Adenocarcinoma - Squamous - Other - Not available | 200 (98.1%)  2 (0.98%)  2 (0.98%)  2 |
| *EGFR* mutation at baseline   - Ex19 deletion - L858R | 137 (66.5%)  69 (33.5%) |
| Responders to osimertinib   - Non responders - Responders - Not available | 44 (22.3%)  153 (77.7%)  9 |
| Type of resistance^2^   - Acquired - Primary Not available | 183 (89.7%) 21 (10.3%) 2 |
| Type of progression^3^   - Multisite - Oligoprogression - Not available | 94 (50.8%)  91 (49.2%)  21 |
| Central nervous system progression   - No - Yes - Not available | 161 (91.0%)  16 (9.0%)  29 |
| Rebiopsy specimen   - Tissue - Plasma - Both - Not available | 133 (66.2%) 37 (18.4%) 31 (15.4%)  5 |

Responders were defined as patients achieving a complete response (CR) or partial response (PR)

^2^ According to Jackman criteria [Jackman et al., J Clin Oncol 2010]

^3^ Multisite progression was defined as disease progression involving more than five sites.

**Supplementary** **Table S3.** Methods used for the detection of MET alterations

| Method | N = 41 (%) | |
| --- | --- | --- |
|  | Tissue | Liquid |
| FISH GCN ≥ 5 | 16 | - |
| NGS^1^ | 5 | 3 |
| IHC | 6 | - |
| FISH GCN ≥ 5 + NGS^1^ | 3 | - |
| FISH GCN ≥ 5 + IHC | 2 | - |
| Not available | 6 | |

^1^ Gene copy number not available

**Supplementary Table S4.** Baseline characteristics of patients who received a second-line therapy.

|  | N=239 (%) |
| --- | --- |
| Age at the time of progression, median (range) | 65 (27-85) |
| Sex   - Female - Male | 157 (65.7%) 82 (34.3%) |
| History of tobacco use   - Ever - Never - Not available | 91 (41.6%)  128 (58.4%)  20 |
| Ethnicity   - White - Asian - Not available | 162 (98.8%)  2 (1.2%)  75 |
| Histology at diagnosis   - Adenocarcinoma - Squamous - Other - Not available | 227 (96.6%)  4 (1.7%)  4 (1.7%)  4 |
| *EGFR* mutation at baseline   - Ex19 deletion - L858R | 145 (60.7%)  94 (39.3%) |
| Responders to osimertinib   - Non responders - Responders - Not available | 55 (22.3%)  171 (77.7%)  13 |
| Type of progression   - Multisite - Oligoprogression - Not available | 113 (54.6%)  94 (45.4%)  32 |
| Central nervous system progression   - No - Yes - Not available | 179 (91.3%)  17 (8.7%)  43 |
| Rebiopsy   - Yes - No | 160 (66.9%) 79 (33.1%) |
| PS ECOG at second-line baseline   - 0-1 - ≥2 - Not available | 182 (80.5%)  44 (19.5%)  13 |
| Second-line treatment based on rebiopsy results   - Yes - No | 39 (16.3%) 200 (83.7%) |

Responders were defined as patients achieving a complete response (CR) or partial response (PR)

^2^ According to Jackman criteria [Jackman et al., J Clin Oncol 2010]

^3^ Multisite progression was defined as disease progression involving more than five sites.

**Supplementary Table S5.** Univariable Cox regression models for PFS.

| Variable |  | Hazard ratio | *p*-value |
| --- | --- | --- | --- |
| **Sex at birth** | Female | Reference |  |
|  | Male | 1.09 (0.82-1.46) | 0.551 |
| **History of tobacco use** | Ever | Reference |  |
|  | Never | 0.9 (0.66-1.21) | 0.476 |
| ***EGFR* mutation at baseline** | Exon 19 deletion | Reference |  |
|  | L858R | 1.15 (0.86-1.53) | 0.350 |
| **Age at progression on osimertinib** | ≥70 years | Reference |  |
|  | <70 years | 1.00 (0.73-1.36) | 0.997 |
| **Type of progression** | Multisite | Reference |  |
|  | Oligoprogression | 0.83 (0.61-1-13) | 0.229 |
| **Central nervous system progression** | No | Reference |  |
|  | Yes | 1.42 (0.83-2-43) | 0.195 |
| **Osimertinib beyond progression** | No | Reference |  |
|  | Yes | 0.78 (0.57-1.07) | 0.119 |
| **ECOG PS at second-line C1D1** | ≥2 | Reference |  |
|  | 0-1 | 0.59 (0.41-0.85) | 0.005 |
| **Rebiopsy at progression** | No | Reference |  |
|  | Yes | 0.69 (0.51-0.93) | 0.016 |
| **Treatment group** | No rebiopsy | Reference |  |
|  | Rebiopsy without tailored treatment | 0.71 (0.51 – 0.93) | 0.03 |
|  | Rebiopsy with tailored treatment | 0.66 (0.44-1) | 0.049 |
| **Center type** | Non academic | Reference |  |
|  | Academic | 1.20 (0.86-1.67) | 0.275 |

**Supplementary Table S6.** Univariable Cox regression models for OS.

| Variable |  | Hazard ratio | *p*-value |
| --- | --- | --- | --- |
| **Sex at birth** | Female | Reference |  |
|  | Male | 1.06 (0.78-1.45) | 0.701 |
| **History of tobacco use** | Ever | Reference |  |
|  | Never | 0.98 (0.71-1.36) | 0.918 |
| ***EGFR* mutation at baseline** | Exon 19 deletion | Reference |  |
|  | L858R | 1.17 (0.86–1.59) | 0.315 |
| **Age at progression on osimertinib** | ≥70 years | Reference |  |
|  | <70 years | 0.87 (0.62-1.22) | 0.422 |
| **Type of progression** | Multisite | Reference |  |
|  | Oligoprogression | 0.69 (0.5-0.97) | 0.032 |
| **Central nervous system progression** | No | Reference |  |
|  | Yes | 1.89 (1.06-3.37) | 0.031 |
| **Osimertinib beyond progression** | No | Reference |  |
|  | Yes | 0.88 (0.68-1-23) | 0.4 |
| **ECOG PS at second-line C1D1** | ≥2 | Reference |  |
|  | 0-1 | 0.45 (0.31-0.67) | <0.001 |
| **Rebiopsy at progression** | No | Reference |  |
|  | Yes | 0.64 (0.46-0.88) | 0.006 |
| **Treatment group** | No rebiopsy | Reference |  |
|  | Rebiopsy without tailored treatment | 0.66 (0.47-0-92) | 0.015 |
|  | Rebiopsy with tailored treatment | 0.6 (0.39-0.93) | 0.021 |
| **Center type** | Non academic | Reference |  |
|  | Academic | 1.12 (0.78-1.61) | 0.544 |

1. Responders were defined as patients achieving a complete response (CR) or partial response (PR)
2. According to Jackman criteria [Jackman et al., J Clin Oncol 2010]
3. Multisite progression was defined as disease progression involving more than five sites.
